# Haploidentical Hematopoietic Stem Cell Transplantation: Expanding the Horizon for Hematologic Disorders

**DOI:** 10.1155/2016/1423493

**Published:** 2016-02-02

**Authors:** Mohammad Faizan Zahid, David Alan Rizzieri

**Affiliations:** ^1^Aga Khan University, Karachi 74800, Pakistan; ^2^Division of Hematologic Malignancies and Cellular Therapy, Duke Cancer Institute, Durham, NC 27710, USA

## Abstract

Despite the advent of targeted therapies and novel agents, allogeneic hematopoietic stem cell transplantation remains the only curative modality in the management of hematologic disorders. The necessity to find an HLA-matched related donor is a major obstacle that compromises the widespread application and development of this field. Matched unrelated donors and umbilical cord blood have emerged as alternative sources of donor stem cells; however, the cost of maintaining donor registries and cord blood banks is very high and even impractical in developing countries. Almost every patient has an HLA haploidentical relative in the family, meaning that haploidentical donors are potential sources of stem cells, especially in situations where cord blood or matched unrelated donors are not easily available. Due to the high rates of graft failure and graft-versus-host disease, haploidentical transplant was not considered a feasible option up until the late 20th century, when strategies such as “megadose stem cell infusions” and posttransplantation immunosuppression with cyclophosphamide showed the ability to overcome the HLA disparity barrier and significantly improve the rates of engraftment and reduce the incidence and severity of graft-versus-host disease. Newer technologies of graft manipulation have also yielded the same effects in addition to preserving the antileukemic cells in the donor graft.

Today's age represents a truly unprecedented time in care of the patient with a hematologic malignancy with the advent of several targeted therapies and novel agents. These therapies have shown tremendous efficacy in treating several malignancies, yielding impressive response rates with limited toxicity profiles [1]. Despite substantial activity against different cancer types, relapse is often encountered at some point during the clinical course of most treated patients, with durable remissions being observed in only a certain percentage of patients. Allogeneic hematopoietic stem cell transplantation (allo-HSCT) is often the only treatment modality which can offer a cure to not only malignant but also benign hematological disorders [2]. A human leukocyte antigen- (HLA-) matched sibling donor is the preferred source of a stem cell graft in allo-HSCT. However, only 30% of patients requiring an allo-HSCT will have an HLA-matched sibling donor [3]. For patients not having a matched sibling donor, a matched unrelated donor (MUD) is an alternate source of stem cells, the probability of finding which is up to 75% among Caucasians. However, these chances are much lower among non-Caucasian populations, with 40% chance in Hispanic individuals and less than 20% among Asian and African American individuals [3–5]. In addition, the cost of recruiting MUDs and maintaining donor registries is a major drawback, making this approach unaffordable and even impractical for developing countries. Hence, it is important to explore other possible stem cell sources.

Unrelated umbilical cord blood (UCB) represents another alternate source of stem cells, one which has been proven to be successful when performing allo-HSCT [6–10]. Key advantages include a comparatively quick donor search and a shorter time to proceed to transplant when compared with adult MUDs [11, 12]. This is especially important in the context of advanced/high-risk hematologic malignancies, where the risk of disease relapse is high and the goal is to proceed to a transplant while the patient is still in remission and has minimal disease burden after induction therapy [13]. In addition, UCB transplantation allows for a greater degree of HLA mismatch, most likely due to a lower number of activated alloreactive T lymphocytes present in cord blood [14, 15]. This accounts for very low and acceptable rates of graft-versus-host disease (GvHD) following UCB transplants [8, 12, 16]. One of the obstacles to UCB transplants is the nonavailability of a sufficient number of hematopoietic progenitor cells in an UCB unit [12]. Although this limitation can be overcome by using more than one unit of cord blood, it is daunted by the high cost of maintaining cord blood banks [17]. Delayed engraftment, increased time to hematopoietic recovery, and immune reconstitution lead to substantially increased risks of life-threatening infections and graft failure, often offsetting the advantages of using unrelated UCB as a stem cell source [5, 12]. Furthermore, due to a limited number of hematopoietic progenitor cells, posttransplant immune manipulation, such as donor lymphocyte infusions, is very difficult, which severely limits treatment options for patients who relapse after transplant [6, 12].

On the other hand, haploidentical related donors offer several key advantages over MUDs and UCB. Almost all patients have at least one haploidentical related donor in the family and the best donor amongst all candidate family members can be selected. Haploidentical related donors are readily available and are highly motivated to donate to a family member [18, 19]. Due to rapid availability of donors and substantially lower costs, patients can proceed to transplant fairly quickly (as early as 3 weeks) [18]. Another major advantage of haploidentical donors over UCB is easy access to the stem cell source that makes donor-derived cellular therapy, such as donor lymphocyte infusions and immune manipulation, available for use after transplant, if necessary [18].

The HLA mismatch between the haploidentical donor and the recipient offers a potent graft-versus-tumor (GvT) effect to completely eradicate malignant cells and offer a permanent cure [18]. Some studies have reported substantially reduced relapse rates with greater degree of HLA mismatches [20–22]. However, two immunological barriers need to be overcome: (1) graft rejection, or host-versus-graft effect, and (2) GvHD. In allo-HSCT studies involving myeloablative conditioning (MAC) regimens, HLA mismatches, either at the antigen level or at allele level, have been associated with inferior survival and poor outcomes after allo-HSCT, with greater degree of mismatch correlating with worse outcomes [21, 23, 24]. Since donor-recipient HLA histocompatibility is the most important independent predictor of outcomes after allo-HSCT, these two barriers are more difficult to overcome in haploidentical hematopoietic stem cell transplantation (haplo-HSCT) than in HLA-matched allo-HSCT. Although the lower risk of relapse due to HLA disparity supports the existence of a GvT effect, this positive aspect of haplo-HSCT is offset by markedly increased rates of GvHD, graft failure, and nonrelapse mortality [23, 25].

Several attempts to perform haplo-HSCT using T lymphocyte replete, unmanipulated grafts have been made. Since donor-derived T lymphocytes are the fundamental players in the pathogenesis of GvHD, these attempts have been associated with early and severe multiorgan failure and GvHD, leading to alarming rates of transplant-related mortality despite pharmacological GvHD prophylaxis [26]. To reduce the risk of GvHD, a new approach of highly purified CD34+ stem cell grafts was adopted using ex vivo T lymphocyte depletion technologies [27]. With this strategy, almost no GvHD was observed in the recipients, despite the absence of posttransplant immunosuppressants for GvHD prophylaxis [28–30]. However, the small number of T lymphocytes transplanted using the highly purified CD34+ stem cell strategy does not allow for an efficient transfer of adoptive immunity, resulting in substantially delayed immune reconstitution and frequent, often fatal, infectious complications [18, 31]. Also, the risk of graft failure is also higher with T lymphocyte depleted haplo-HSCT [27, 32].

There is a key difference between positive selection of CD34+ progenitor cells and negative depletion of lymphocytes during graft manipulation. With CD34+ selection strategies, almost no cells other than the CD34+ stem cells are transplanted. On the other hand, negative depletion techniques (mainly CD3+ and CD19+ negative depletion) allows for other types of cells, such as dendritic cells, natural-killer (NK) cells, and monocytes, to be retained in the donor graft and transplanted with the CD34+ progenitors. Both graft manipulation techniques result in effective removal of B and T lymphocytes, leading to greatly reduced risks of developing posttransplant Epstein-Barr virus related lymphoproliferative disorders (B lymphocyte depletion) and GvHD (T lymphocyte depletion) [26, 33]. The detailed research and broad application of graft manipulation has provided insight into the biology of NK cells, lymphocytic constituents of the innate immune system, and their immunotherapeutic potential in allo- and haplo-HSCT [34].

NK cell activity is modulated by the interaction between killer cell immunoglobulin-like receptor (KIR) expressed on the surface of NK cells and cognate ligands (i.e., certain HLA alleles) to KIRs [35]. Engagement of KIRs by their corresponding ligands produces inhibitory signals and prevents NK cell activation. In the context of allo-HSCT, and especially haplo-HSCT, NK cells in the allograft can attack malignant cells in the recipient if there is a mismatch between the KIRs on the donor-derived NK cells and the HLA antigens on the recipient (and malignant) cells [36]. With so many haploidentical donors available for a vast majority of patients, the ideal donor can be picked based on the KIR haplotype on the donor NK cells to exploit their alloreactivity and induce powerful GvT effects [37, 38]. Additionally, the disparity between KIRs and HLA haplotypes does not increase the risk of developing GvHD [39].

Another fundamental difference between CD34+ selection and negative depletion of lymphocytes (CD3+/CD19+ depletion) is that the number of alloreactive lymphocytes “contaminating” the donor graft is approximately ten times higher in CD3+/CD19+ depletion. This necessitates the use of pharmacological GvHD prophylaxis after transplants using CD3+/CD19+ depleted grafts [33, 40]. However, a notable problem with the transplantation of T lymphocyte depleted grafts is the increased likelihood of relapse after transplantation [41, 42]. A more efficient approach is the depletion of T-cell receptor (TCR) *αβ*+ T lymphocytes and CD19+ cells (TCR*αβ*+/CD19+ depletion) from donor grafts [43, 44]. This approach greatly increases the efficiency of alloreactive T lymphocyte removal (similar to that of CD34+ selection techniques), while retaining other cells such as NK cells and monocytes. In addition, this method also retains *γδ*+ T lymphocytes in the graft. *γδ*+ T lymphocytes constitute approximately 5% of circulating T lymphocytes. These cells are nonalloreactive and exhibit potent antitumor and anti-infectious properties [5, 44]. Since these cells are not activated by major histocompatibility complex (MHC) interactions, they do not react to alloantigens and hence do not contribute to GvHD [45, 46]. This is advocated by the fact that patients exhibiting high *γδ*+ T lymphocyte counts after allo-HSCT show better survival with reduced incidence of relapse and GvHD [47]. In contrast, the majority of circulating T lymphocytes (approximately 95%) are *αβ*+ T lymphocytes which act against alloantigens via MHC interactions and give rise to GvHD after allo-HSCT [48]. The TCR*αβ*+/CD19+ depletion technique allows for satisfactory removal of *αβ*+ T lymphocytes to prevent GvHD while retaining *γδ*+ T lymphocytes to ensure timely immune reconstitution and a robust GvT effect, especially after haplo-HSCT [47, 49, 50]. The most recent study [51] reporting the results of haplo-HSCT with TCR*αβ*+/CD19+ depletion in pediatric patients showed acceptable rates of GvHD (22%). In all but one patient, GvHD was grade 2 and responded to first-line therapy. Graft failure was observed in 27% of patients, all of whom were successfully retransplanted with different rescue protocols. The overall survival of patients from this study was 96.7%, advocating the efficacy of TCR*αβ*+/CD19+ depletion in improving outcomes after haplo-HSCT.

During the 1980s, outcomes of haplo-HSCT were discouraging due to severe GvHD and graft failure, often approaching rates as high as 90% [52, 53]. The introduction of graft manipulation and T lymphocyte depletion brought about notable improvements in outcomes of recipients of haplo-HSCT by dramatically reducing the incidence of GvHD [54]; however, the high incidence of graft failure remained a major obstacle to the application of haplo-HSCT [55, 56]. Graft failure is mediated by native cytotoxic T lymphocytes that persist in the recipient's system even after conditioning regimens are administered [57]. This barrier in histocompatibility was shown to be overcome in several animal-model studies [58] and, subsequently, in clinical studies [57, 59, 60] by infusing high doses of T lymphocyte depleted hematopoietic progenitor cells. Additional strategies to help engraftment included enhancing the ablative intensity of conditioning regimens and addition of anti-T lymphocyte agents [57, 59, 61]. Unfortunately, such intensive conditioning regimens result in extensive organ damage and toxicities which limit their use to younger and relatively healthier patients in comparison to individuals of older age groups [5]. Chemotherapeutic doses and total body irradiation used in MAC regimens cause extensive damage to host tissue and release of inflammatory cytokines, such as tumor necrosis factor alpha (TNF*α*) and interferon gamma (IFN*γ*). These inflammatory mediators, and several others, play an integral role in the pathophysiology of GvHD [62], which may be one of the reasons why MAC regimen transplants are frequently complicated by GvHD [57, 63]. The problem with T lymphocyte depleted grafts is delayed immune reconstitution and significant period of immunodeficiency which predisposes to serious and often fatal infections [28, 57, 64].

Nonmyeloablative conditioning (NMAC) regimens are an attractive alternative for patients requiring an allo-HSCT who are not considered suitable candidates for MAC regimens due to advanced age, comorbidities, and/or increased risks of therapy-related adverse effects [65]. Using NMAC regimens also results in the decreased release of inflammatory mediators and cytokines and may consequently reduce the incidence and magnitude of GvHD, especially when used for haplo-HSCT. This significantly broadens the applicability of transplantation to patients who would otherwise be unfit to receive a transplant using standard protocols. Earlier studies applying haplo-HSCT with NMAC regimens showed discouraging patient outcomes, especially with alarming rates of graft failure [66, 67]. Rizzieri et al. [68] published the first large study on haplo-HSCT with fludarabine, cyclophosphamide, and alemtuzumab used as the preparatory NMAC regimen which demonstrated that the complications of GvHD and graft failure after haplo-HSCT could be overcome, making it a feasible treatment option in the therapy of patients with hematologic malignancies. 75% of the patients in this study achieved complete remission with only 10.2% transplant-related mortality in the first 100 days. Engraftment rate was high, with only 14% of patients suffering from graft failure, while only 8% of patients developed GvHD. However, relapse and infectious complications were major causes of mortality in this study (49% and 22%, resp.). One plausible reason for the high rate of relapse could be that the patients participating in this study harbored poor-risk hematologic malignancies which carry an inherent probability of relapse.

Cyclophosphamide is a highly immunosuppressive alkylating agent with an established role in anticancer chemotherapy and HSCT [69]. Historically, cyclophosphamide is commonly administered as part of several conditioning regimens prior to HSCT; however, as previously discussed, the cytotoxic effects of this agent lead to tissue damage and release of inflammatory mediators and increase the risk of GvHD [62]. Administration of cyclophosphamide between 48 and 72 hours after transplant has been shown to facilitate posttransplant immune-tolerance; however, this tolerance is not induced when the drug is administered at 24 or 96 hours after transplant [70]. Cyclophosphamide exploits the high cytotoxic sensitivity of alloreactive T lymphocytes (both host and donor) to DNA damage [71]; hence carefully timed administration of posttransplant cyclophosphamide inhibits GvHD and graft rejection [72, 73]. O'Donnell et al. [69] investigated the role of posttransplantation cyclophosphamide in patients receiving haplo-HSCT after NMAC conditioning. The results of their study concluded that this strategy substantially reduced the risks of GvHD and graft rejection and was able to produce long-lasting donor-recipient chimerism after transplant. Subsequently, Luznik et al. [64] reported their experience with using posttransplant cyclophosphamide after NMAC haplo-HSCT. The results of their study showed acceptable rates of graft failure (13%) and GvHD (grades 2–4: 34%, grades 3-4: 6%) with rapid achievement of donor-recipient chimerism after transplant. Administration of two doses of cyclophosphamide in the 48-to-72-hour window was associated with a lower risk of chronic GvHD than only one dose, further advocating that posttransplant cyclophosphamide is efficacious in inducing posttransplant tolerance. However, relapse of primary disease was a major cause of treatment failure and mortality (up to 51%). This study also recruited patients with poor-risk hematologic malignancies, which may explain the high incidence of relapse in this study group. Patients with lymphoid malignancies were at a lower risk of experiencing relapse than those with myeloid malignancies, indicating particular effectiveness of cyclophosphamide in treating lymphoid hematologic malignancies. Other studies have demonstrated similar findings, corroborating that this strategy depletes alloreactive T lymphocytes in the donor graft as well as the host immune system, dramatically reducing the risk of both GvHD and graft failure, which are much more difficult to control in the setting of haplo-HSCT and are substantial impediments [69]. While there is concern that posttransplant cyclophosphamide might damage or kill donor hematopoietic progenitors cells, these cells have high expression of aldehyde dehydrogenase enzymes which confer relative resistance to the cytotoxic effects of the drug [74].

A relatively newer strategy to increase the success rates of haplo-HSCT is the use of T regulatory cells (Tregs). Tregs are a specialized subset of CD4+ lymphocytes with concurrent expression of CD25 and FOXP3 genes which play crucial roles in antitumor immunity, posttransplant tolerance, and protection against autoimmunity [75–77]. In addition, they have low expression of CD127 [78]. The adoptive transfer of these specialized T lymphocytes limits graft and host alloreactivity, leading to reduced risks of GvHD and graft failure [79–82]. Tregs also preserve and maintain normal lymph node and thymus architecture from GvHD and allow for a quick posttransplant immune recovery while having no negative effects on the GvT responses of allo-HSCT [83]. Additional studies have also showed that Tregs become activated and proliferate by interaction with recipient antigen-presenting cells in the proinflammatory milieu after administration of conditioning regimens, attenuating alloreactive T lymphocyte activation while allowing nonalloreactive T lymphocyte expansion to ensure a timely immune recovery [82, 84–86].

Di Ianni et al. [87] performed the first human study evaluating the adoptive transfer of Tregs on posttransplantation outcomes in patients receiving haplo-HSCT. Early infusion of donor-derived Tregs on day −4 of transplant, followed by infusion of positively immunoselected CD34+ stem cells and conventional T lymphocytes on day 0, showed prevention of GvHD in the absence of any immunosuppressive GvHD prophylaxis and hastened posttransplant immune recovery and immunity against opportunistic pathogens and did not have any negative effects on GvT effects [87]. A follow-up study of these patients [84] showed successful engraftment in 26/28 (93%) patients. Only 2 (7.7%) of 26 evaluable patients developed grade 2–4 GvHD. Spectratyping analysis showed the rapid development of T lymphocyte repertoire within months after transplant. Naïve and memory T lymphocyte subpopulations showed rapid increase over the first year after transplant, while CD4+ and CD8+ lymphocytes against opportunistic pathogens like* Aspergillus* and* Toxoplasma* appeared significantly earlier in comparison to historical controls comprising standard haplo-HSCT recipients. At a median follow-up of 21 months, 46.1% were alive and in remission [84].

Tregs can be isolated in vitro using standard donor-derived leukapheresis products using double negative selection with anti-CD8 and anti-CD19 monoclonal antibodies and positive selection for CD25 [88]. Expression of the FOXP3 gene represents the peripheral mature Tregs [89], while low CD127 expression correlates with exhibition of regulatory functions in this T lymphocyte subset [90]. Such cell-based transplantation and graft modifications prove to improve posttransplantation engraftment, allow quick immune reconstitution, and reduce GvHD; however, the expertise required for such cellular manipulations limits their clinical implementation to highly specialized medical centers.

With regard to Tregs, posttransplant GvHD prophylaxis may also play a role in selectively favoring the expansion of Tregs while suppressing mature effector T lymphocytes. Sirolimus is an immunosuppressant that demonstrates such properties [91]. Peccatori et al. [92] performed a recent study showing that a calcineurin inhibitor-free, sirolimus-based GvHD prophylaxis regimen promoted selective Treg expansion after haplo-HSCT. This strategy was well tolerated and a robust and rapid engraftment was observed in the majority of patients. While the incidence and severity of acute and chronic GvHD in this study were comparable to historical data from MUD allo-HSCT using peripheral blood stem cells [93, 94], they were much higher when compared with haplo-HSCT using bone marrow as the source of donor stem cells [64, 95, 96]. In addition, Tregs show a higher level of resistance to the cytotoxic effects of cyclophosphamide [97]. Future studies may be performed to ascertain the specific effects of GvHD prophylaxis in T lymphocyte dynamics to systematize the utility of regimens based on their effects on T lymphocyte dynamics to derive the maximum benefit, carefully weighing GvHD, GvT, engraftment, and immune recovery after transplant.

In conclusion, allo-HSCT remains the only treatment modality that offers a potential cure for some with hematologic disorders. HLA-matched donors are not always available, which has led to establishment of UCB and MUDs as alternative sources for stem cell transplantation. However, the high cost of maintaining donor registries and cord blood banks, limited size of the cord blood units, and high-risk nature of the ablative procedure limit the expansion of these donor sources as well. HLA haploidentical donors are an attractive choice for stem cells since nearly every patient has an available haploidentical donor from their family. In modern medicine, new strategies such as “megadose stem cell infusions” and posttransplantation immunosuppression with cyclophosphamide have shown the ability to overcome graft failure and GvHD, complications that occurred at overwhelmingly high rates and severely limited the application of haploidentical transplantation before the 21st century. Other strategies such as NMAC regimens prior to transplant and pretransplant graft manipulation have demonstrated similar survival rates when compared with HLA-matched allo-HSCT with MAC regimens, but with significantly reduced toxicities associated with HSCT and preservation of the antileukemic properties of immune cells in the donor graft. Future prospective studies to explore the biology of the GvT effects in this setting can provide more perspective on the advantages and drawbacks of haplo-HSCT over “traditional” allo-HSCT, suggest further enhancements of the process, and move this “now-controversial” modality into the standard of care for patients with limited options.
